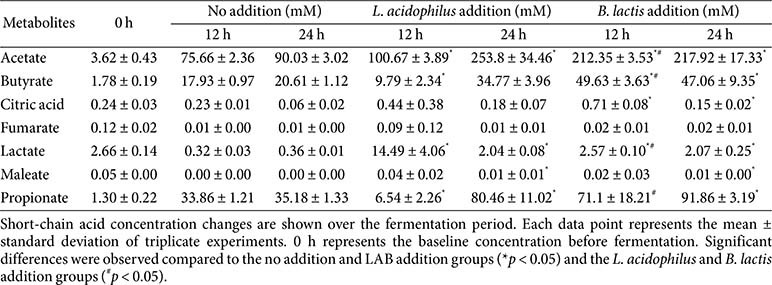# Gastrointestinal Tolerance and Gut Microbiota Modulation of Encapsulated and Free Forms of *Lactobacillus acidophilus* and *Bifidobacterium animalis* subsp. *lactis*

**DOI:** 10.4014/jmb.2506.06028

**Published:** 2025-09-24

**Authors:** Geonhee Kim, Hyunbin Seong, Seung Hee Han, Hwa Rin Kim, Sung-Hwan Kim, Hye-Jin Ku, Hye-Jung Han, Chul-Hong Kim, Nam Soo Han

**Affiliations:** 1 Brain Korea 21 Center for Smart GreenBio Convergence and Sustainable Regional Development, Chungbuk National University, Cheongju 28644, Republic of Korea; 2 Division of Animal, Horticultural, and Food Science, Chungbuk National University, Cheongju 28644, Republic of Korea; 3 Food Research Center, Binggrae Co. Ltd., Namyangju 12253, Republic of Korea

**Keywords:** Probiotic encapsulation, gastrointestinal survivability, *in vitro* digestion and fecal fermentation, gut microbiota and metabolites

## Abstract

The viability and efficacy of probiotics are strongly influenced by their delivery form, especially under harsh gastrointestinal conditions. This study assessed the gastrointestinal resistance of encapsulated and free from *Lactobacillus acidophilus* and *Bifidobacterium animalis* subsp. *lactis*, and evaluated the specific effects of digested free from strains on the gut microbiome. An *in vitro* digestion model simulating gastric and intestinal conditions was used to assess probiotic survival, followed by fecal fermentation to examine microbial and metabolic responses. The encapsulated probiotics, formulated with a multilayer matrix, demonstrated significantly higher viability and preserved membrane integrity than the free forms. Despite reduced viability, free from probiotics modulated the gut microbiota. Both strains promoted colonization of beneficial genera such as *Bifidobacterium*, *Lactobacillus*, and *Prevotella*, while maintaining microbial balance without inducing dysbiosis. Additionally, probiotic supplementation enhanced short-chain fatty acid production, particularly acetate and butyrate, indicating increased microbial fermentation activity. Notably, distinct ecological patterns emerged between the two strains, with *L. acidophilus* inducing dynamic shifts and recovery, and *B. lactis* contributing to structural stability. These findings underscore the importance of strain-specific selection and encapsulation strategies in developing targeted and effective probiotic interventions.

## Introduction

Probiotics are defined as live microorganisms that, when consumed in adequate amounts, provide health benefits to the host [1]. They support epithelial integrity, regulate immune responses, and produce beneficial metabolites such as short-chain fatty acids (SCFA) [2]. To exert their health benefits, probiotics must survive the harsh physicochemical conditions of the gastrointestinal (GI) tract, which include gastric acidity, bile salts, digestive enzymes in the small intestine, and physical or oxidative stress during transit. However, many strains lose significant viability under such conditions, diminishing their probiotic activity when consumed [3, 4].

The health-promoting effects of probiotics have been extensively documented. These strains have been shown to enhance GI tolerance, modulate the gut microbiota, and stimulate the production of SCFAs [5]. Since the functional efficacy of these probiotics is already well recognized, recent studies have increasingly focused on improving their survival and delivery through the gastrointestinal tract, particularly in the context of functional foods and supplements. To enhance probiotic survival, encapsulation technologies have been developed to protect cells during digestive transit [6]. Various encapsulation methods, including extrusion, emulsion, spray drying, and soft gel, utilize materials such as alginate, gelatin, chitosan, and lipid [7]. Early systems with single-layer coating provided basic protection but were vulnerable to gastrointestinal conditions [8]. In contrast, recent approaches based on multilayer encapsulation have demonstrated improved performance by combining biopolymers and lipids to enhance the physical and chemical stability of probiotic formulations [9]. Materials such as pectin and gelatin have been used for their buffering capacity and gel-forming ability, while soy lecithin can stabilize microbial membranes and vegetable oil may introduce a hydrophobic barrier that reduces acid and enzyme penetration [10]. Although the protective potential of each of these materials has been examined individually, their combined use in a multilayer encapsulation system has not been extensively explored.

Most studies have evaluated probiotic viability under simplified static acidic or intestinal conditions, but often were unable to distinguish live, dead, or membrane-compromised (damaged) cells and did not assess interactions with gut microbiota, thereby limiting understanding of their ecological contributions [11, 12]. Moreover, many studies have often focused on single probiotic strains, making it difficult to compare their impacts on gut microbiota and limiting comprehensive understanding of probiotic effects [13]. To address these gaps, it is necessary to use advanced analytical techniques and experimental systems. Confocal laser scanning microscopy (CLSM) is used to evaluate cell membrane integrity and viability [14]. CLSM combined with fluorescent staining distinguishes live, dead, and damaged cells, enabling comparison of encapsulation strategies and understanding of damage levels [15]. In addition, a precision-controlled *in vitro* fecal fermentation model assesses interactions between probiotics and gut microbiota [16]. This system closely simulates gut conditions and allows dynamic control of parameters such as pH, oxygen levels, and nutrient supply, enabling real-time observation of the gut ecosystem [17]. High-throughput techniques, such as metagenome sequencing and metabolite analysis, provide insights into probiotics’ ecological and metabolic impacts [18]. These integrated approaches form a solid foundation for understanding probiotic survival, colonization, and function in the gut.

This study aimed to compare the gastrointestinal survivability of encapsulated versus free forms of *Lactobacillus acidophilus* and *Bifidobacterium lactis* and investigate their strain-specific effects on gut microbiota composition and short-chain fatty acid production using an *in vitro* digestion and fecal fermentation model. For this purpose, the survivability of each strain in both encapsulated and free form was assessed using viable cell counts and CLSM analysis during simulated *in vitro* digestion. Subsequently, an *in vitro* fecal fermentation model was conducted using probiotic strains in free form after digestion to eliminate the potential confounding effects of encapsulation materials on the gut microbiota. Microbial composition was analyzed using 16S rRNA metagenome sequencing, and the production of metabolites, including SCFA, was quantified using proton nuclear magnetic resonance spectroscopy.

## Materials and Methods

### Microorganisms and Materials

Two probiotic strains, *L. acidophilus* NCFM and *B. animalis* subsp. *lactis* Bl-04, were used in encapsulated and free (non-encapsulated) form. The encapsulated form of the strains was commercially manufactured by Morishita Jintan Co., Ltd. (Japan). According to the manufacturer’s Certificate of Analysis, two capsule formulations were used, each specifically designed for each probiotic strain. The *L. acidophilus* capsule contained vegetable oil, fish gelatin, glycerin, soy lecithin, pectin, and *L. acidophilus*. The *B. lactis* capsule consisted of vegetable oil, fish gelatin, glycerin, potato starch, soy lecithin, pectin, and *B. lactis*. All capsules were stored at 4°C in sealed containers and used without further modification prior to experimentation. The free form of strains was obtained as lyophilized powders from Danisco (Denmark). Pepsin (from porcine gastric mucosa, 3,200–4,500 U/mg protein), pancreatin (from porcine pancreas), bile salts, CaCl_2_·2H_2_O, KH_2_PO_4_, NaCl, NaHCO_3_, KCl, MgCl_2_·6H_2_O, (NH_4_)_2_CO_3_, and glucose were purchased from Sigma-Aldrich (USA). Peptone, yeast extract, and hemin were obtained from BD Difco (USA). Vitamin K_1_ was obtained from Wako (Japan).

### Enumeration Method

To recover viable cells from the capsule for enumeration, 5 g of encapsulated probiotic was hydrated in PBS, vortexed for 30 min, and homogenized using a bench-top homogenizer for 30 sec. The disrupted sample was further decapsulated with pancreatin at 37°C for 2 h to dissolve residual coating. The homogenate was centrifuged at 10,000 ×*g* for 5 min, and bacterial pellets were recovered for plating and viability assessment. For the free form, enumeration was performed directly without a decapsulation step, whereas the encapsulated form underwent the decapsulation process described above prior to enumeration. Viable bacterial counts were performed using serial dilutions on Tryptic Soy (TS) agar (Sigma-Aldrich), De Man, Rogosa, and Sharpe (MRS) agar (BD Difco), and TOS-MUP (Sigma-Aldrich) for total anaerobes, lactic acid bacteria, and *Bifidobacterium* spp., respectively.

### *In Vitro* Digestion

Simulated gastrointestinal digestion was performed according to a previously reported protocol [19]. Encapsulated (10 g) or free probiotics (1 g) were suspended in 20 ml PBS. For gastric digestion, the samples were mixed in equal volume (1:1, v/v) with simulated gastric fluid (SGF), composed of 0.5 g/l KCl, 0.12 g/l KH_2_PO_4_, 2.1 g/l NaHCO_3_, 2.76 g/l NaCl, 0.02 g/l MgCl_2_·6H_2_O, 0.04 g/l (NH_4_)_2_CO_3_, and 0.02 g/l CaCl_2_·2H_2_O. The pH was adjusted to 3.0 using 1 M HCl. Pepsin was added at a final concentration of 2,000 U/ml, along with CaCl_2_·2H_2_O (final concentration; 0.3 mM), and the mixture was incubated at 37°C for 2 h in a shaking incubator at 150 rpm. For small intestinal digestion, 40 ml of simulated intestinal fluid (SIF) was prepared, consisting of 0.5 g/l KCl, 0.1 g/l KH_2_PO_4_, 7.14 g/l NaHCO_3_, 2.24 g/l NaCl, and 0.07 g/l MgCl_2_·6H_2_O. Equal volume (1:1, v/v) of SIF was added to the post-gastric mixture. The pH was adjusted to 7.0 using 1 M NaOH. 100 U/ml Pancreatin and 10 mM bile salts were added, and the mixture was incubated under the same conditions (37°C, 150 rpm) for 2 h. All digestion procedures were conducted under strict anaerobic conditions inside a vinyl anaerobic chamber (Coy Laboratory Products Inc., USA), maintained with a gas composition of 85% N_2_, 10% CO_2_, and 5% H_2_.

### Confocal Microscopy for Cell Integrity

The structural viability of probiotic cells before and after simulated digestion was assessed using LIVE/DEAD BacLight bacterial viability staining (SYTO9/PI, Invitrogen, USA). Cells were collected, washed with saline, and stained for 15 min in the dark. Confocal images were acquired using a Zeiss LSM 880 (Zeiss, Germany) with a 100 × oil immersion objective. Green fluorescence indicated viable cells, red indicated dead cells, and orange/yellow indicated membrane-compromised, damaged cells. Image analysis was performed using Zeiss LSM Image Browser software.

### *In Vitro* Fecal Fermentation

*In vitro* human fecal fermentation of digested probiotics was performed following a previously established protocol [17]. Digested free probiotics were centrifuged at 10,000 ×*g* for 1 min at 4°C, and the supernatant was discarded. The bacterial pellet was resuspended in 50 ml of prepared basal growth medium. Fecal samples were obtained from healthy adult donors (three males and two females, aged 25–35 years) who had no history of gastrointestinal disorders and had not taken antibiotics within the past three months. Donors followed a typical Korean diet and had not consumed probiotic supplements. A 10% (w/v) fecal slurry was prepared by homogenizing fresh feces in PBS and diluted in the fermentation vessel to achieve a final fecal concentration of 1% (v/v) in the basal medium. The basal medium consisted of glucose (10 g/l), peptone (2 g/l), yeast extract (1 g/l), bile salts (0.5 g/l), vitamin K1 (10 μl/l), hemin (50 mg/l), and buffering salts. Digested probiotic suspensions were added to achieve a final concentration of 10^8^ CFU/ml, and the mixtures were incubated anaerobically at 37°C for 24 h. Samples were collected at 0, 12, and 24 h for microbiota and metabolite analysis. The Institutional Review Board of Chungbuk National University approved the study protocol and consent form IRB No. 2023024431.

### 16S rRNA Metagenome Analysis

Microbial community profiles in the fecal fermentation samples were characterized through high-throughput sequencing of the 16S rRNA gene using the Illumina MiSeq platform (Illumina, USA). The hypervariable V3–V4 regions were amplified using the primer pair 341F (5'-CCTACGGGNGGCWGCAG-3') and 785R (5'-GACTACHVGGGTATCTAATCC-3'), compatible with the Nextera indexing system [19]. Raw sequence data were demultiplexed, trimmed for quality using Cutadapt, and processed using the Deblur algorithm in the QIIME2 pipeline [20, 21]. Taxonomic classification of amplicon sequence variants was performed with a pre-trained Naive Bayes classifier based on the SILVA 132 reference database (https://www.arb-silva.de/documentation/release-132/). Alpha diversity metrics, including observed operational taxonomic units and the Shannon index, were computed to assess within-sample diversity. Analyses were conducted in a reproducible conda-based QIIME2 environment. Relative abundance shifts following probiotic treatment were calculated by comparing genus-level proportions across timepoints.

### Metabolite Profiling via ^1^H-NMR

Metabolite profiling of the fermented samples was carried out using proton nuclear magnetic resonance (^1^H-NMR) spectroscopy, as described previously with minor modifications [22]. After *in vitro* fecal fermentation, samples were centrifuged at 16,000 ×*g* for 10 min to separate the supernatant. The clarified supernatant was diluted with an equal volume of deionized water containing 10% (v/v) D_2_O and 1 mM sodium 2,2-dimethyl-4-silapentane-1-sulfonic acid (DSS), resulting in a final DSS concentration of 0.5 mM. The pH was adjusted to 6.00 ± 0.01 using 2 M HCl or NaOH. A 700 μl aliquot of each sample was transferred into 5-mm NMR tubes (Norell, USA). Spectra were acquired on a Varian INOVA 500 MHz NMR spectrometer (Varian Inc., USA). Metabolite identification and quantification were performed using the Processor and Profiler modules of the Chenomx NMR Suite version 6.1 (Chenomx Inc., Canada).

### Statistical Analysis

Each experiment was conducted in triplicate, and data are presented as the mean ± standard deviation. Statistical analyses were performed using IBM SPSS software version 22 (SPSS Inc., USA) to assess significant differences. Independent t-tests were used to compare two groups: *L. acidophilus* addition vs. no addition, *B. lactis* addition vs. no addition, and *L. acidophilus* addition vs. *B. lactis*. One-way ANOVA was independently conducted within each probiotic treatment group (*L. acidophilus* and *B. lactis*) to assess changes in the relative abundance of health-related microbiota over time. A significance level of *p* < 0.05 was used.

## Results

### Viability of Probiotics under Simulated Gastrointestinal Conditions

**Fig. 1:**
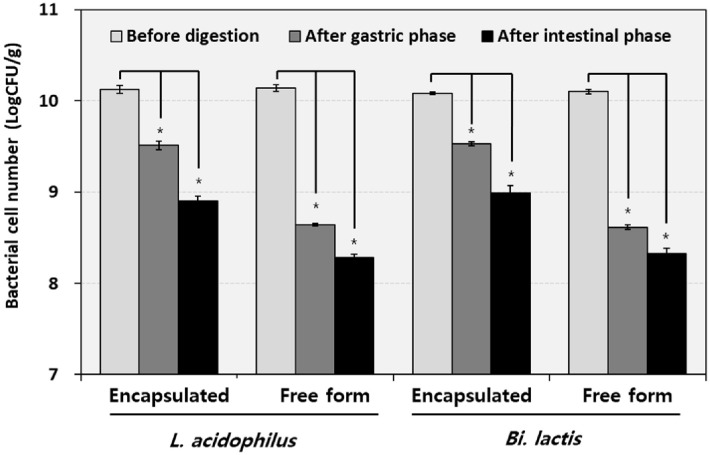
Survival of probiotics in different formulations (encapsulated and free forms) after passage through the *in vitro* human gastrointestinal tract. Probiotic viability (Log CFU/g) was measured at three stages: Before digestion refers to samples not exposed to digestive fluids, representing baseline counts. After the gastric phase indicates samples treated with simulated gastric fluid (pH 3.0, with pepsin) for 2 h. After the small intestinal phase denotes subsequent treatment with simulated intestinal fluid (pH 7.0, containing bile salts and pancreatin) for an additional 2 h. Error bars represent the standard deviation of triplicates. Significant differences were determined by comparing with the initial bacterial counts (*p < 0.05).

To evaluate the protective effect of encapsulation, both encapsulated and free forms of *L. acidophilus* and *B. lactis* were subjected to an *in vitro* digestion model simulating oral, gastric, and intestinal phases (Fig. 1). Both encapsulated strains exhibited significantly higher resistance to acidic and enzymatic stress than their free form (non-encapsulated). Encapsulated *L. acidophilus* decreased from 10.13 to 9.51 Log CFU/g after the gastric phase and to 8.90 Log CFU/g after the intestinal phase, showing an approximately 15-fold decrease from the initial count. In contrast, the free form dropped from 10.14 to 8.64 and 8.28 Log CFU/g, indicating an approximately 79-fold decrease from the initial count. Similarly, encapsulated *B. lactis* declined from 10.08 to 9.53 and 8.99 Log CFU/g, representing an approximately 12-fold decrease from the initial count, whereas the free form decreased from 10.10 to 8.61 and 8.33 Log CFU/g, showing an approximately 59-fold decrease from the initial count. Overall, *L. acidophilus* experienced slightly greater viability loss than *B. lactis* in both formulations, highlighting strain-specific differences in stress tolerance. These results support the beneficial effects of encapsulation during gastrointestinal transit, even under conditions of pH 3.0.

### Cell Integrity and Survivability of Probiotic during *In Vitro* Digestion

**Fig. 2:**
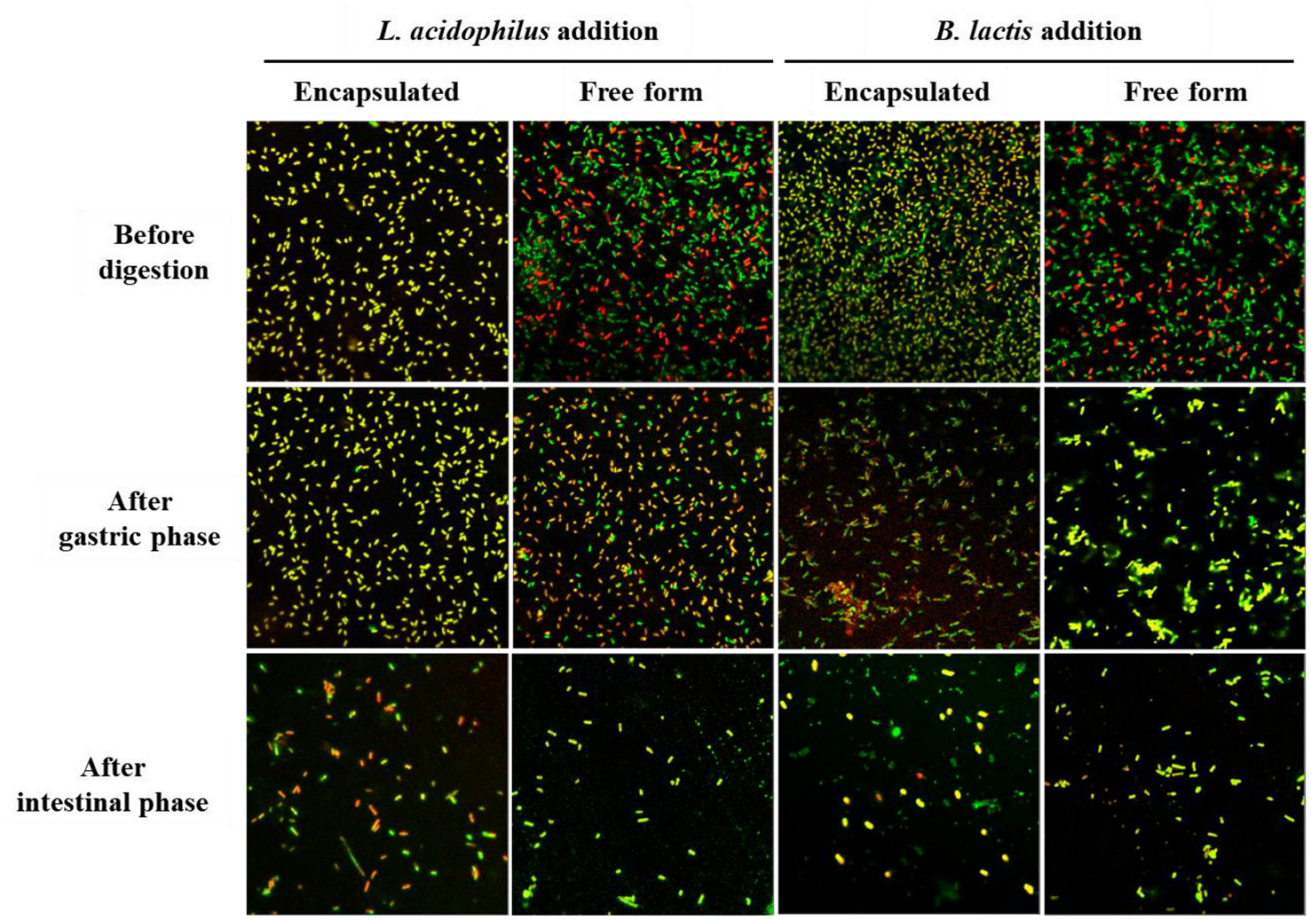
Confocal laser scanning microscopy images showing the viability of *L. acidophilus* and *B. lactis* in different formulations during simulated gastrointestinal digestion. Samples were collected at three stages of *in vitro* digestion: before digestion, after the gastric phase, and after exposure to simulated intestinal conditions. Each row represents a different probiotic formulation. Bacterial viability was assessed using LIVE/DEAD staining: SYTO9 stains live cells green, propidium iodide (PI) stains dead cells red, and cells with compromised membranes appeared yellow or orange due to overlap of both signals. A gradual reduction in bacterial counts across the digestion stages was observed, due to bacterial dilution during the simulated gastrointestinal transit.

Structural integrity was assessed by confocal microscopy with LIVE/DEAD staining (SYTO9 stained all cells green, PI stained damaged cells red, and yellow/orange indicated partial membrane damage). Encapsulation enhanced probiotic survival and integrity during gastrointestinal transit (Fig. 2). For *L. acidophilus*, the encapsulated form showed yellow/orange fluorescence before digestion, indicating many partially permeabilized cells. After gastric stress, this pattern largely persisted with high viable cell proportions and few dead cells, and intestinal digestion showed similar results. In contrast, the free form exhibited mixed green/red fluorescence before digestion, with increased damaged cells and decreased partially permeabilized cells after gastric digestion, and a predominance of dead cells after intestinal digestion. For *B. lactis*, the encapsulated form similarly displayed yellow/orange fluorescence before digestion, indicating partial membrane damage but high viability. After gastric stress, it maintained this favorable state, and after intestinal digestion, viable cells remained high while dead cells were low. The free form showed mixed green and red fluorescence before digestion, with increased damaged cells after gastric digestion and some remaining after intestinal digestion. These findings confirm encapsulation’s protective effect, supporting its role in enhancing probiotic viability and delivery efficiency through the gut.

### Effects of Free Probiotic Supplementation on Gut Microbiota Composition

**Fig. 3:**
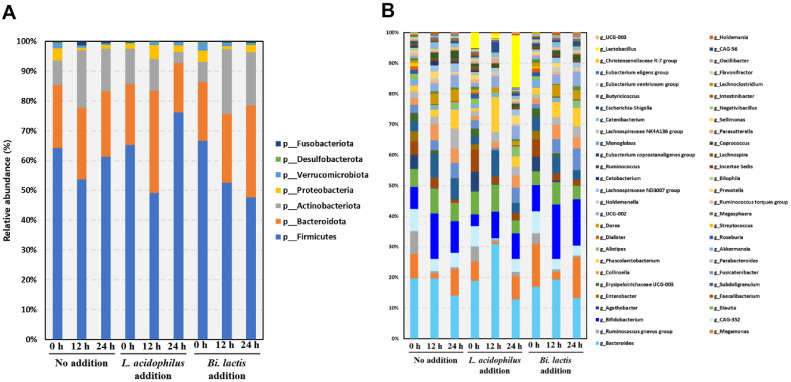
Effects of free form *L. acidophilus* and *B. lactis* on gut microbiota composition during *in vitro* fecal fermentation. (**A**) Microbial composition at the phylum level and (**B**) at the genus level after 0, 12, and 24 h of *in vitro* fecal fermentation. Fermentation was conducted using fecal slurry from 5 healthy human donors, with samples collected at 0 (baseline), 12, and 24 h for metagenomic analysis. Three groups were compared: no addition (control), addition of *L. acidophilus*, and addition of *B. lactis*.

The influence of free form *L. acidophilus* and *B. lactis* on gut microbiota was assessed over a 24 h fecal fermentation period using 16S rRNA metagenome sequencing. Accurate evaluation of the strain-specific effects of *L. acidophilus* and *B. lactis* was achieved by using only free form probiotics in fecal fermentation experiments, following *in vitro* digestion, thereby facilitating a direct and unbiased assessment of their impacts on the gut microbial ecosystem. At the phylum level (Fig. 3A), the no addition group showed typical profiles, with Firmicutes increasing to 61.21%, Bacteroidota at 22.00%, and Actinobacteriota slightly decreasing to 14.27%. During the 24 h *in vitro* fecal fermentation, *L. acidophilus* supplementation increased Firmicutes from 65.25 ± 0.85% to 76.17 ± 4.99%, and reduced Bacteroidota from 20.44 ± 0.85% to 16.52 ± 5.00%, and Actinobacteriota from 11.78 ± 0.4% to 3.66 ± 1.14%. While *B. lactis* supplementation reduced Firmicutes from 66.60 ± 2.84% to 47.63 ± 18.13% and increased Actinobacteriota and Bacteroidota from 6.74 ± 1.87% and 17.82 ± 1.10% to 17.72 ± 6.16 and 30.86 ± 10.94%, respectively. At the genus level (Fig. 3B), during the 24 h *in vitro* fecal fermentation with *L. acidophilus* supplementation, the relative abundance of *Lactobacillus* increased from 4.62 ± 0.01% to 16.29 ± 0.08%, and *Megamonas* rose from 5.74 ± 0.08% to 7.08 ± 0.56%, whereas *Bacteroides* decreased from 17.39 ± 0.41% to 12.37 ± 0.52%. *B. lactis* supplement increase *Bifidobacterium* from 8.02 ± 0.30%to 14.22 ± 0.38% and increased *Phascolarctobacterium* from 2.04 ± 0.73% to 5.72 ± 0.23%, with *Bacteroides* decreasing from 16.01 ± 0.74% to 12.54 ± 1.65%. Collectively, *L. acidophilus* supplementation led to an increase in *Lactobacillus* and *Megamonas*, whereas *B. lactis* supplementation promoted the growth of *Bifidobacterium* and *Phascolarctobacterium*, also contributed to moderate changes in *Bacteroides*. These results indicate that probiotic supplementation induced distinct shifts in gut microbial composition, and a detailed continuous analysis was conducted to further understand these changes.

### Microbiome Diversity Analysis

**Fig. 4:**
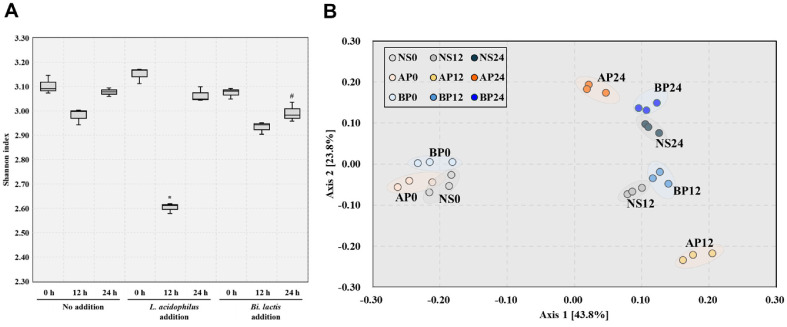
Microbial diversity and compositional shifts during *in vitro* fecal fermentation with probiotic supplementation. (**A**) Alpha diversity measured by the Shannon index across fermentation time points (0, 12, and 24 h) for each treatment group. (**B**) Principal coordinates analysis (PCoA) based on Bray–Curtis dissimilarity showing microbial changes during *in vitro* fecal fermentation. NS, no addition; AP, *L. acidophilus* addition; BP, *B. lactis* addition; 0 h represents the baseline diversity before fermentation. 12 and 24; after 12 and 24 h *in vitro* fecal fermentation. Significant differences were observed compared to the no addition and *L. acidophilus* addition group (*p < 0.05) and *B. lactis* addition group (#*p* < 0.05).

The influence of probiotic supplementation on gut microbial diversity was assessed by monitoring changes over a 24 h *in vitro* fecal fermentation period. In the *L. acidophilus* group, the Shannon index transiently decreased at 12 h and increased again by 24 h, although it remained lower than both the control and the *B. lactis* group (Fig. 4A). In contrast, the *B. lactis* group maintained alpha diversity levels comparable to the control throughout the fermentation period, with a slight decline to a similar level as the control at 24 h (Fig. 4A). This pattern suggests that probiotic treatment initially stimulated ecological activation but did not induce long-term disruption of microbial diversity. To evaluate changes in microbial community composition over time, beta diversity was assessed using PCoA with Bray–Curtis dissimilarity. The resulting plots showed distinct clustering patterns corresponding to each probiotic treatment and time point. (Fig. 4B). Notably, by 24 h, samples in the *L. acidophilus* and *B. lactis* groups formed distinct but non-divergent clusters, indicating strain-specific effects while preserving the general trajectory of community adaptation. These findings suggest that probiotic addition transiently reprograms the gut microbiota in a controlled and balanced manner, maintaining overall community dynamics without deviating from the ecological path observed in non-supplemented conditions.

### Changes in Health-Related Gut Microbiome

An analysis of changes in health-related gut microbiota was conducted using delta (Δ) values, which account for baseline variation, to determine the fine-scale impact of probiotic supplementation on gut microbiota composition. These Δ values represent the changes in relative abundance at 24 h of fermentation, corrected by shifts observed in the no addition group. Specifically, Δ values were calculated as follows:

Δ = (Abundance at 24 h in *L. acidophilus* or *B. lactis* addition group - Abundance at 0 h in *L. acidophilus* or *B. lactis* addition group) - (Abundance at 24 h in no addition group - Abundance at 0 h in no addition group)

The analysis targeted microbiota known to influence gut health, based on previous classification systems [23]. In total, 49 health-positive (H+) and 46 health-negative (H−) microbiota were included along with 19 probiotic strains approved by the Korean Ministry of Food and Drug Safety (Table S1). Of these, 16 H+ and 11 H− microbiota were detected in our fecal samples. Table 1 presents the 27 microbiota detected and their abundance changes. Δ values of gut microbiota were used to assess microbiome shifts after 24 h of *in vitro* fecal fermentation, adjusting for baseline fluctuations from the no addition group. In the *L. acidophilus* addition group, *L. acidophilus*, the H+ microbiota, showed the most significant increase in relative abundance (Δ +10.58 ± 0.83%), followed by a significant increase in *Prevotella copri* (Δ +1.37 ± 0.37%). In the H− group, *Bacteroides coprocola* showed a significant increase (Δ +1.36 ± 0.00%), while *Bacteroides vulgatus* exhibited a significant decrease (Δ −0.79 ± 0.32%). In the *B. lactis* supplementation group, *B. lactis* demonstrated a significant increase in relative abundance (Δ +5.89 ± 1.05%) followed by a significant increase in *Bifidobacterium catenulatum* (Δ +0.76 ± 0.17%). In the H− group, *Bacteroides fragilis* (Δ +1.05 ± 0.43%) showed a significant increase. In summary, supplementation with *L. acidophilus* and *B. lactis* promoted colonization of the administered probiotic strains, supported the presence of key health-associated microbiota, and maintained overall microbial stability.

### Production of Microbial Short-Chain Fatty Acids during Fecal Fermentation

Microbial metabolite production was evaluated at 0 and 24 h following *in vitro* fecal fermentation with free probiotic supplementation (Table 2). Short-chain fatty acids (SCFAs) were quantified using ^1^H-NMR spectroscopy, which revealed that both probiotics significantly enhanced SCFA production compared to the control. At 24 h, *L. acidophilus* addition resulted in the highest total SCFA concentrations (253.8 ± 34.46 mM acetate, 80.46 ± 11.02 mM propionate, 34.77 ± 3.96 mM butyrate), followed by *B. lactis* addition (217.92 ± 17.33 mM acetate, 91.86 ± 3.19 mM propionate, 47.06 ± 9.35 mM butyrate), whereas the control group yielded lower levels (90.03 ± 3.02 mM acetate, 35.18 ± 1.33 mM propionate, 20.61 ± 1.12 mM butyrate). The accumulation of lactate remained modest, while citric acid and fumarate levels declined compared with baseline, suggesting microbial utilization. These results indicate robust fermentative activity by both strains. While acetate was dominant in all groups, *L. acidophilus* produced the highest total SCFA, whereas *B. lactis* contributed more to propionate and butyrate accumulation. Collectively, these findings demonstrate that the two strains distinctly modulate fermentation profiles and enhance SCFA production through strain-specific metabolic mechanisms.

## Discussion

This study assessed the gastrointestinal survivability of encapsulated *L. acidophilus* and *B. lactis* using an *in vitro* digestion model and investigated the influence of free form strains on the gut microbial ecosystem by analyzing microbiota composition and metabolite production through *in vitro* fecal fermentation. The study highlighted the gastrointestinal survival and protective effect conferred by encapsulation. The significant protective effects of the encapsulation system were demonstrated in Fig. 1, highlighting that despite digestive stress, encapsulated *L. acidophilus* and *B. lactis* maintained high levels of viability. In contrast, free form of probiotics experienced substantial reductions in viable cell counts during simulated gastrointestinal digestion. This enhancement in survival is attributed to the encapsulation matrix, which is a multilayer soft gel system composed of vegetable oil, fish gelatin, glycerin, potato starch, soy lecithin, and pectin, enclosing the probiotic core (based on manufacture’s Certificate of Analysis document).

This encapsulation matrix provided an effective physical barrier that shields the probiotic cells from low pH and bile salt exposure [3]. Even under the harsher condition of pH 1.2, it has been reported that, despite differences in bacterial strains, free form bacterial powder is more likely to lose viability, further emphasizing the effectiveness of encapsulation [24]. The functional properties of the encapsulation materials further support their role in GI protection. Fish gelatin forms a hydrogel matrix that preserves probiotic viability under acidic and enzymatic conditions by acting as a stabilizing scaffold [25]. Soy lecithin enhances membrane integrity and improves probiotic survival in low pH and bile salt environments [26]. Vegetable oil provides a hydrophobic barrier that reduces exposure to gastric fluids and digestive enzymes [27], while pectin contributes buffering capacity and improves acid resistance [28]. These materials synergistically enhance the resilience of probiotics against gastrointestinal stress. These findings highlight encapsulation as a crucial strategy for improving the delivery and viability of probiotics in the gastrointestinal tract.

Confocal microscopy revealed structural heterogeneity between encapsulated and free form cells after *in vitro* digestion phase. Encapsulated strains exhibited a higher proportion of orange-fluorescent cells suggestive of sublethal membrane injury, while free form strains showed more pronounced red fluorescence, indicating greater membrane disruption (Fig. 2). This observation highlights that encapsulation protects membrane integrity and mitigates stress-induced disruption during gastrointestinal transit, which is essential for effective probiotic formulations [3, 29].

Differential modulation of gut microbiota and fermentation by probiotic strains was observed. Following inoculation of *L. acidophilus*, the abundance of *Lactobacillus* initially showed a transient decline, possibly due to adaptation stress in the host environment, but subsequently increased over time, suggesting successful colonization. The administration of *B. lactis* led to a prompt increase in *Bifidobacterium* levels, which remained elevated throughout the observation period, indicating stable colonization and ecological resilience. This pattern is consistent with previous studies showing the most ingested probiotics transiently alter microbial interactions [30, 31]. In the *L. acidophilus* addition group, *Lactobacillus* levels increased at 24 h, and notably, *Bifidobacterium* maintained its relative abundance despite the overall tendency to decline observed in the no addition group. This stability may reflect the activation or support of resident bifidobacteria via lactate-mediated cross-feeding, whereby lactate produced by *Lactobacillus* facilitates the growth of *Bifidobacterium* and butyrate-producing microbes [32]. In the *B. lactis* addition group, the overall microbial composition remained relatively stable between 12 and 24 h, showing a consistent taxonomic profile with minimal temporal shifts. This contrasts with the *L. acidophilus* group, where compositional changes were more pronounced over the same period. The relative stability observed in the *B. lactis* group suggests that the introduced bifidobacterial strain may have supported ecological resilience without perturbing the resident microbiota. Notably, both *Bifidobacterium* and *Bacteroides* were consistently detected at comparable levels, which may reflect a balanced microbial community structure. Previous studies have highlighted the importance of such configurations, where *Bacteroides*, as dominant saccharolytic commensals [33], can generate fermentation intermediates that potentially support *Bifidobacterium* activity through metabolic complementarity or cross-feeding interactions [34]. These findings suggest that *B. lactis* administration may reinforce existing microbial networks, contributing to the maintenance of microbial homeostasis in the gut.

In addition to the direct effects of viable probiotic cells, some components of the encapsulation matrix may also influence gut microbial dynamics. Although the present fermentation experiments were conducted using free from probiotics, in actual consumption scenarios, encapsulated formulations may interact with the gut microbiota through their matrix components. For instance, pectin is known to act as a fermentable prebiotic that selectively promotes the growth of *Bifidobacterium* and *Faecalibacterium*, contributing to mucosal health and SCFA production [35]. Vegetable oils have been associated with altered microbial activity by serving as metabolic substrates for lipid-utilizing bacteria like *Bacteroides* [36]. Thus, beyond their protective function during gastrointestinal transit, these encapsulating materials may also contribute to shaping the gut microbiota *in vivo*.

Interestingly, although *L. acidophilus* supplementation induced more pronounced compositional shifts and a sharper decline in microbial diversity at 12 h, it also led to a more complete recovery by 24 h. Conversely, *B. lactis* supplementation, while associated with relatively stable community structure, resulted in only partial restoration of diversity. These contrasting patterns suggest that *L. acidophilus* may have engaged the gut ecosystem through dynamic resilience, characterized by a capacity for rapid reorganization and recovery following perturbation. In contrast, *B. lactis* may exemplify structural stability, integrating smoothly with minimal ecological disruption but exhibiting slower re-diversification. This distinction reflects findings from previous studies showing that probiotic interventions often induce a transient decrease in microbial diversity followed by spontaneous recovery, indicating short-term ecological activation and re-equilibration [37]. The restoration of diversity toward baseline levels without prolonged disruption further supports the notion of transient but balanced interactions with the resident microbiota, underscoring the gut ecosystem’s inherent adaptability and resilience. Together, these findings highlight that both *L. acidophilus* and *B. lactis* exert modulatory effects without disrupting the resident microbiota, a feature that aligns with key goals of probiotic intervention: ecological compatibility and support of host microbial homeostasis.

Health-positive (H+) and health-negative (H−) bacterial changes in the fecal microbiome were specifically investigated in this study to evaluate the beneficial effects of ingested probiotics on gut microbial balance. Chang *et al*. (2024) conducted a comparative analysis of 8,069 fecal shotgun metagenomes collected from 54 studies involving both healthy individuals and patients with various diseases. The study identified 49 H+ and 46 H-bacterial species that effectively discriminated between healthy and diseased conditions. This classification achieved a balanced cross-validation accuracy of 80%, demonstrating the robustness of these microbial indicators. In the present study, we also examined the presence and modulation of these health-related bacteria in our *in vitro* fecal fermentation samples following treatment with two probiotic strains. To expand the scope of health-positive taxa, we supplemented the original H+ list with 19 probiotic strains approved for use in Korea. As a result, a curated pool of 68 H+ and 46 H− species was established and used for comparative metagenomic analysis (Table S1). As shown in Table 1, 16 H+ species and 11 H− species were detected among the fecal microbiota across all samples. In the *L. acidophilus* and *B. lactis* addition groups, two H+ species and one H− species showed significant increases. Furthermore, the relative abundances of the administered probiotic strains increased notably in both groups (*L. acidophilus*, Δ10.58 ± 0.83%; *B. lactis*, Δ4.09 ± 0.78%), indicating successful adaptation to the gut environment and promoting colonization and microbial stability. In the *L. acidophilus* group, *Prevotella copri* exhibited a significant increase in relative abundance. The metabolites produced by *L. acidophilus*, particularly lactate, may serve as growth substrates for *P. copri*, facilitating its proliferation and enhancing carbohydrate metabolism and SCFA production [38]. These metabolic effects contribute to improved gut barrier integrity and immune modulation, suggesting that *L. acidophilus* supplementation may support gut health [39]. In the *B. lactis* group, *Bacteroides fragilis* also showed a significant increase in relative abundance. Although certain toxin-producing strains of *Ba. fragilis* are associated with inflammatory diseases and colorectal cancer [40], non-toxigenic strains are generally considered beneficial commensals that contribute to gut homeostasis [41]. Thus, the observed increase in *Ba. fragilis* in this study may reflect a beneficial ecological shift, supporting microbial balance without inducing pathogenic effects. Overall, both probiotic strains facilitated colonization of the administered microbes and helped maintain a stable balance of health-related gut taxa. These findings underscore their potential to enhance gut health through targeted and functionally meaningful modulation of the gut microbiome. The selective impact on H+ and H− microbial populations provides further insight into the functional consequences of *L. acidophilus* and *B. lactis* supplementation.

SCFA production increased with probiotic supplementation in this study. In the *L. acidophilus* group, total SCFA levels, especially acetate and butyrate, increased, while lactate accumulated modestly and citric acid and fumarate levels decreased. In the *B. lactis* group, total SCFA levels also rose, though less markedly, with acetate as the predominant metabolite. These findings highlight the distinct yet complementary metabolic profiles of the two probiotic strains. As integrated outputs of microbial metabolism, SCFA concentrations serve as sensitive indicators of probiotic functionality, providing insights into microbial activation and host-relevant outcomes [42]. *L. acidophilus* primarily engages in homolactic fermentation to generate lactate and acetate, which may support the growth of other beneficial taxa through cross-feeding interactions and, in turn, enhance epithelial integrity and improve metabolic function [33]. Meanwhile, *B. lactis* produces acetate and propionate via the fructose-6-phosphate phosphoketolase pathway and the succinate pathway [43]. Acetate and propionate produced by *B. lactis* can enhance barrier integrity, reduce gut inflammation, and modulate host immune responses [44]. In addition to their direct effects on the host, the SCFAs generated by *L. acidophilus* and *B. lactis* can serve as key metabolic substrates for other microbial taxa within the gut, fostering microbial cross-feeding interactions and promoting the growth of saccharolytic bacteria. Both strains appeared to enhance microbial metabolic interactions by providing fermentation byproducts, creating favorable conditions that supported saccharolytic activity [45]. The SCFA production patterns observed reflect the probiotic-driven remodeling of the gut’s metabolic environment, underscoring their complementary roles in enhancing gut metabolic function and supporting fermentative balance.

While this study provides valuable insight into formulation-dependent differences in probiotic survivability and gut microbiota interactions, a couple of limitations should be acknowledged. First, encapsulated probiotics were intentionally excluded from the fecal fermentation phase to preserve experimental integrity and isolate each strain’s metabolic contributions. Using encapsulated probiotics in the fermentation phase could introduce confounding factors, as capsule materials may act as additional carbon sources or alter microbial metabolism, thereby masking strain-specific effects. Therefore, we used only free forms to directly attribute observed metabolic changes to probiotic activity. Nevertheless, based on the enhanced gastrointestinal resistance of encapsulated probiotics observed in this study, they are expected to have stronger modulatory effects on gut microbiota than the free form tested under experimental conditions, during actual consumption. Next, confocal analysis of encapsulated probiotics offers the advantage of clearly illustrating the condition of each probiotic cell step by step, making it an advanced analytical technique compared to previous encapsulation studies. For this analysis, the pre-treatment process involves complete disintegration of the capsule to obtain images that distinguish live, dead, and damaged cells. This pre-treatment is also essential for quantifying the viable cell counts of encapsulated probiotics after their passage through the stomach and small intestine. After a series of comparative experiments, homogenizing treatment followed by pancreatic enzyme treatment, as described in the experimental methods, was selected as the most effective method. However, analysis showed that this treatment resulted in a decrease of approximately 20% in viable cell counts, which likely affected the study results (data not shown). To achieve more accurate comparative studies of viable encapsulated probiotics, the development of a capsule disintegration technique that does not affect cell viability is required in the future.

## Conclusion

This study investigated how probiotic formulation affects both gastrointestinal survivability and modulation of gut microbiota. Using a standardized *in vitro* digestion and fecal fermentation system, we compared encapsulated and free form of *L. acidophilus* and *B. lactis*. Encapsulated probiotics exhibited superior survivability under simulated digestive conditions, while free forms, despite lower viability, effectively modulated microbial composition by increasing beneficial taxa and SCFA production. Specifically, *L. acidophilus* acted as a microbial activator promoting beneficial metabolites, whereas *B. lactis* contributed to microbiome stability. Although this study focused on digested free-form probiotics during fermentation, the enhanced survivability of encapsulated strains under simulated digestive conditions suggests their potential functional relevance *in vivo*. Importantly, free form probiotics still demonstrated significant modulatory effects on microbial composition and SCFA production. These findings underscore the importance of both formulation strategy and strain selection in probiotic design. Future research should expand to *in vivo* validation and host-level analyses to guide targeted probiotic development.

## Supplemental Materials

Supplementary data for this paper are available on-line only at http://jmb.or.kr.



## Figures and Tables

**Table 1:** Quantitative delta values of gut microbiota taxa classified as health-associated (H+) or healthnegative (H−) following the addition of *Lactobacillus acidophilus* or *Bifidobacterium lactis* addition.

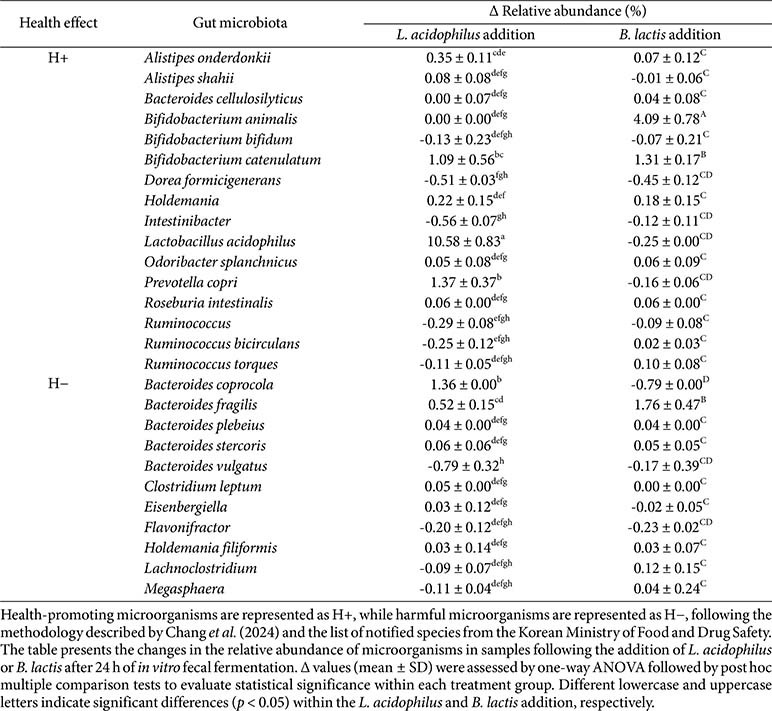

**Table 2:** Changes in short-chain acids concentration (mM) during *in vitro* fecal fermentation after 12 and 24 h.