# Supplementary material for: Gastrointestinal Tolerance and Gut Microbiota Modulation of Encapsulated and Free Forms of Lactobacillus acidophilus and Bifidobacterium animalis subsp. lactis
Source: J Microbiol Biotechnol. 2025 Sep 24;35:e2506028. doi: 10.4014/jmb.2506.06028 (PMC12535859; doi:10.4014/jmb.2506.06028)
Supplement: Supplementary file 1 [file jmb-35-e2506028-supple.pdf]

J. Microbiol. Biotechnol. 2025. 35: e2506028

https://doi.org/10.4014/jmb.2506.06028

Supplementary Figure

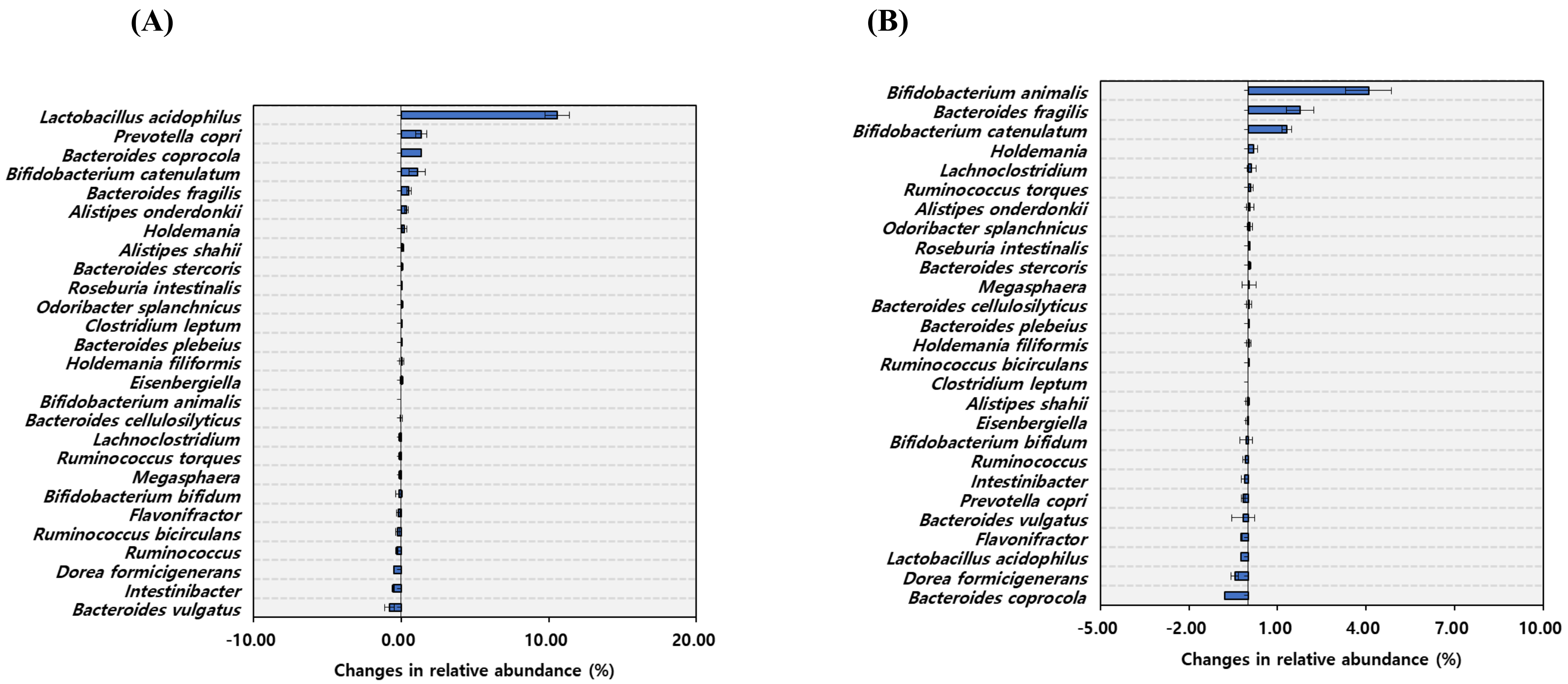

Fig. S1. Changes in core health-related gut microbes after 24 h of in vitro fecal fermentation with (A) *L. acidophilus* and (B) *Bi. lactis* addition. Microbial changes in relative abundance are expressed as delta values calculated as follows: (abundance of individual microorganisms in the sample with *L. acidophilus* or *Bi. lactis* addition at 24 h − abundance of individual microorganisms in the sample with *L. acidophilus* or *Bi. lactis* addition at 0 h) − (abundance of individual microorganisms in the no addition sample at 24 h − abundance of individual microorganisms in the no addition sample at 0 h). **(A)** represents samples with *L. acidophilus* addition, and **(B)** represents samples with *Bi. lactis* addition during in vitro fecal fermentation.
